# Domain wall pinning in FeCoCu bamboo-like nanowires

**DOI:** 10.1038/srep29702

**Published:** 2016-07-11

**Authors:** Eider Berganza, Cristina Bran, Miriam Jaafar, Manuel Vázquez, Agustina Asenjo

**Affiliations:** 1 Instituto de Ciencia de Materiales de Madrid, CSIC, Madrid, 28049, Spain

## Abstract

The three dimensional nature of cylindrical magnetic nanowires has opened a new way to control the domain configuration as well as the magnetization reversal process. The pinning effect of the periodic diameter modulations on the domain wall propagation in FeCoCu individual nanowires is determined by Magnetic Force Microscopy, MFM. A main bistable magnetic configuration is firstly concluded from MFM images characterized by the spin reversal between two nearly single domain states with opposite axial magnetization. Complementary micromagnetic simulations confirm a vortex mediated magnetization reversal process. A non-standard variable field MFM imaging procedure allows us to observe metastable magnetic states where the propagating domain wall is pinned at certain positions with enlarged diameter. Moreover, it is demonstrated that it is possible to control the position of the pinned domain walls by an external magnetic field.

The development of devices based on spintronics attracts much interest due to its advantages in electronics regarding non-volatility, reduced power consumption and increased data processing speed^1^.

Particularly, the control of the magnetic domain wall (DW) motion is a key aspect for the functionalization of ferromagnetic-based devices, logic systems or sensing devices^2^,^3^,^4^,^5^. A large number of works are currently devoted to the study of DW dynamics along ferromagnetic elements driven by electric current^6^,^7^,^8^ or magnetic fields^9^,^10^. In order to develop DW-based applications, one has to address some fundamental questions about the DW configuration and the reversal mechanism. Both features play a major role in the propagation speed of the DWs, amongst other parameters. On the other hand, the understanding of pinning/depinning mechanism becomes essential for the use of well-localized artificial pinning sites that enable the trapping of DWs at selected positions.

The most widespread method of creating pinning centers in nanostripes is patterning notches with different shapes in planar nanostructures^11^,^12^,^13^. However, individual cylindrical nanowires have attracted less attention^14^,^15^,^16^, despite presenting some features that make them more convenient for applications, such as not showing a Walker breakdown^14^,^17^ that limits the DW propagation speed at high external fields. It is also worth noticing that their cylindrical geometry favors the development of vortex domain walls that move uniformly^10^, contrary to the transverse domain walls extensively studied in two dimensional structures^18^,^19^. Thus, it can be of great interest the control over the nucleation and positioning of domain walls in this nanowires for a number of alternative logic and 3D magnetic storage devices, such a race-track memory, where bits are coded as magnetic domain walls along each wire^3^,^11^,^20^. An additional advantage of these nanostructures is the low-cost technique used for the fabrication.

In this study, ferromagnetic cylindrical nanowires with negligible crystalline anisotropy and very high aspect ratio have been investigated. Their longitudinal uniaxial magnetic anisotropy makes them ideal systems to study the magnetization reversal process. A significant work has been devoted to the preparation of ferromagnetic cylindrical nanowires grown into templates by electrochemical route^21^,^22^. For this work, cylindrical nanowires with periodically distributed small segments of different diameters –labeled as bamboo like NWs- were prepared, as well as NWs of continuous diameter- straight NWs.

CoFe based alloy nanowires have been selected due to their large saturation magnetization and high Curie temperature, which makes them good candidates to replace rare-earth free based permanent magnets in certain applications.

The final aim of this study is to determine the local magnetic configuration along individual nanowires and to show the pinning effect of the bamboo-like geometry in FeCoCu nanowires making use of an advanced Magnetic Force Microscopy (MFM) technique^23^,^24^. MFM is a recognized powerful technique to image the local magnetization configuration^25^ as well as the magnetization reversal process at the nanoscale^26^. This technique provides high resolution images of the magnetic configuration (around 20nm) together with the corresponding topographic information. MFM images have been firstly obtained at remanence and under an external applied magnetic field parallel to the cylinder axis. To gain deeper understanding of the magnetization reversal process, a more subtle imaging procedure has been used. Complementary micromagnetic simulations were carried out using object orientated micromagnetic framework^27^ (OOMMF) package to confirm the experimental results.

## Results and Discussion

### Determining the effect of the modulation on the spin configuration

Nanowires with very high aspect ratio were grown by electrodeposition into the pores of anodic alumina membranes. Modulated pores are produced by pulsed hard anodization in oxalic aqueous solution. By suitable tuning of the electrochemical parameters we were able to introduce periodical changes into the diameter in a controlled way^28^.

**Figure 1:**
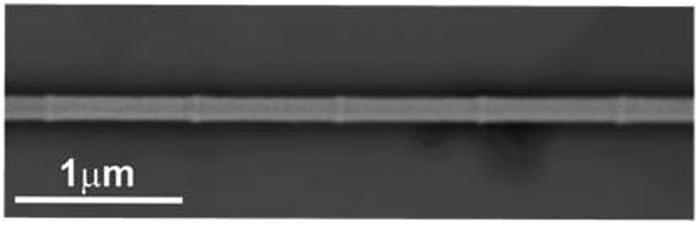
Scanning electron microscopy image of an individual bamboo-like nanowire.

The total length of the nanowires is about 12 μm. They display a bamboo-like structure with a periodicity of 800 nm and with diameters of about 150 nm (segment) and 170 nm (modulation), respectively (See Fig. 1). The composition, determined by Energy Dispersive Spectroscopy (EDS), of the nanowires alloy is Fe_28_Co_67_Cu_5_ (hereafter referred to as the FeCoCu nanowires).

**Figure 2:**
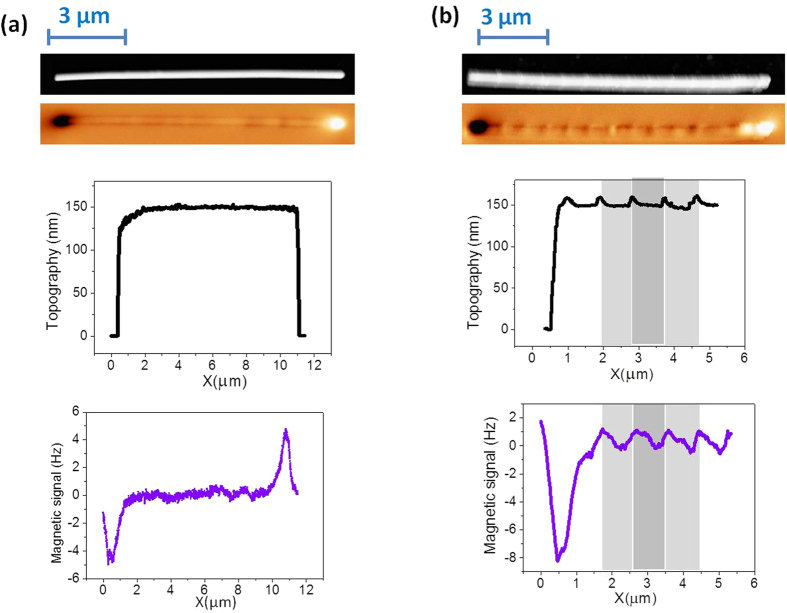
Topographic and MFM images of (**a**) a NW with homogeneous diameter showing a single domain configuration and (**b**) a bamboo-like NW where the local modulations produce small divergences of the magnetization. Profiles along the main axis were performed in both NWs (only left part of the bamboo-like NW is shown for clarity purposes).

Homogeneous and bamboo-like NWs with similar length were studied. Nanowires of constant diameter (φ = 150 nm) were used as a reference to evaluate the effect of the diameter modulation. A few tens of homogeneous and bamboo-like NWs were imaged in remanence after ac demagnetization. In most of the cases, images like those shown in Fig. 2 are observed. The magnetic image in Fig. 2a corresponds to the homogeneous diameter nanowire, where the dark and bright contrasts at the ends of the NW are the main noticeable features. That leads us to conclude its single domain configuration.

However, the MFM image in Fig. 2b, corresponding to the bamboo NW, displays the same dark and bright contrast at the ends plus additional, less intense, periodic contrasts along the wire. The comparison between magnetic and topographic images demonstrates the correlation between the periodic modulations in the diameter and the features in the MFM contrast.

**Figure 3:**
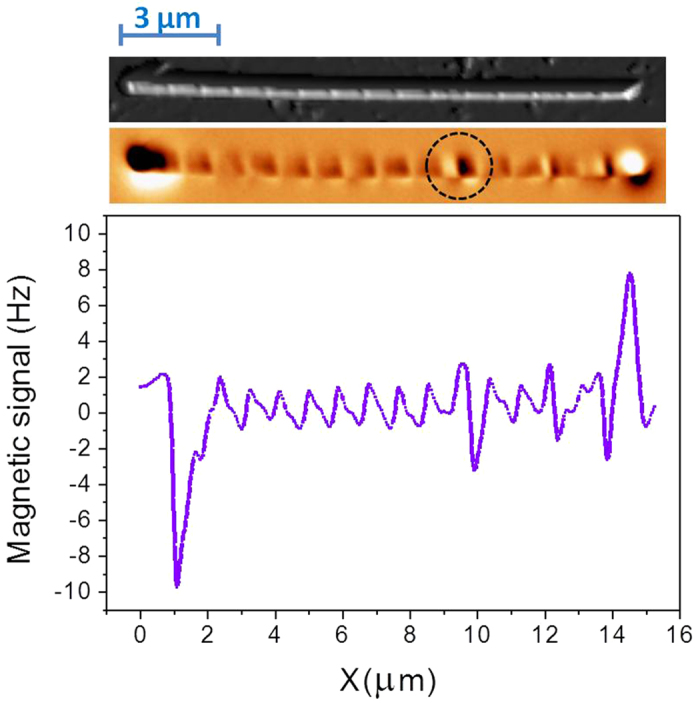
Nanowire with several strong black-white contrasts in the positions of enlarged diameter.

Although the configuration shown in Fig. 2b is the most usual, we have also found different behaviors. In the MFM image in Fig. 3, high contrast (bright and dark) spots are identified in certain positions that match with enlarged diameter regions along the NW (marked with a circle). Despite not been energetically favorable, such strong contrast could be eventually interpreted as originated by a small domain with the magnetization opposite to the main nanowire magnetization.

In summary, most of the bamboo-like nanowires (as shown in Fig. 2b) present MFM images that are compatible with the single domain configuration with a periodic local spin divergence induced at the sites of increased diameter. On the other hand, the presence of a vortex (or a system of vortices) is expected in this kind of nanowires. In order to obtain deeper insight into the magnetic configuration and their evolution under applied magnetic field, an advanced variable field MFM technique in combination with micromagnetic simulations has been applied.

**Figure 4:**
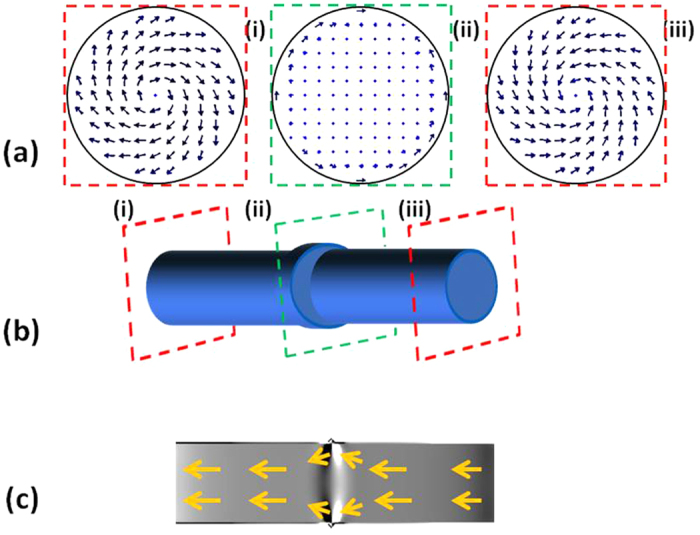
Micromagnetic simulations of the spin configuration of a modulated NW calculated by OOMMF. Different vortex configurations (**a**) appear along the NW (i), (ii) and (iii). (**b**) Sketch of the simulated nanowire. (**c**) In a section along the wire, magnetization divergence plus some arrows indicating the magnetization direction are depicted.

The micromagnetic simulations corresponding to the bamboo-like nanowires (see Fig. 4) reveal that the expected spin configuration is a combination of a vortex -where the spins follow a circular path at the enlarged diameter shape- plus a configuration with the core spins aligned with the cylinder axis. The simulated spin configuration displays two equal vortices appearing at both edges of the NW (i) and (iii). However, at the position with enlarged diameter (ii) a pseudo-vortex with a large core is expected. Notice that the MFM is hardly sensitive to this pseudo-vortex due to the lack of stray field. Nevertheless, the bright and dark contrasts along the NW axis are induced by the weak stray field close to the modulation. Similar contrast appears also in the simulated MFM image shown in Fig. 4c that corresponds to the divergence of the magnetization.

### Imaging the magnetization reversal process

Magnetization reversal processes in both homogeneous and bamboo nanowires were investigated by using a non-standard MFM technique^24^ whose major asset is its capability to image the magnetization reversal process of individual elements.

In this MFM based mode, a profile along the main axis of the desired nanowire is repeatedly scanned while an *in-situ* external magnetic field -up to +/−70mT-is swept.

In modulated NWs, the in-plane magnetic fields, parallel to the main axis of the NWs, are not high enough to reach the magnetic saturation, but are sufficient to reverse their magnetization. Further details are supplied in the Supplementary Information 1.

**Figure 5:**
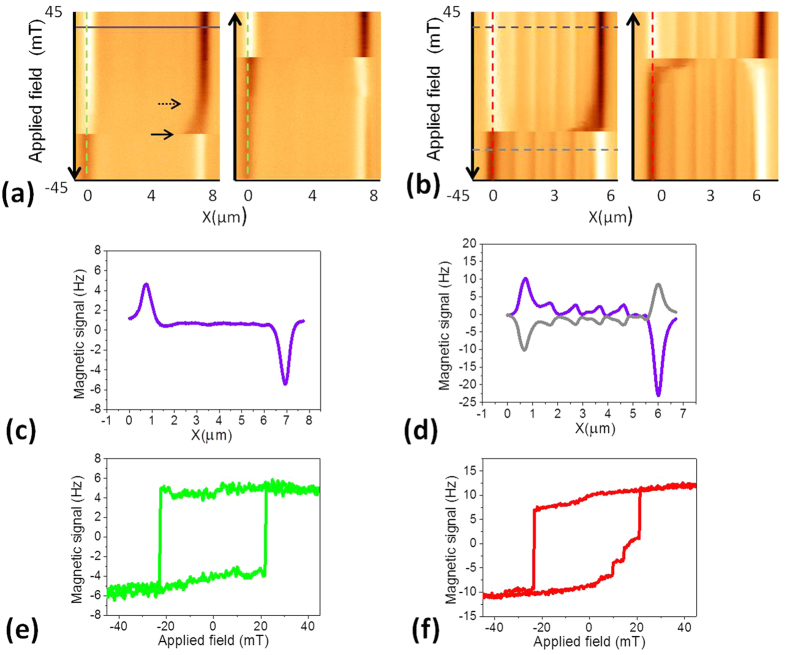
Non-standard MFM based image of nanowires (**a**) with homogeneous diameter and (**b**) bamboo-like geometry. (**a**) Two critical fields have been marked with arrows. (**b**) In the bamboo-like nanowires, a DW propagated towards the opposite end, jumping from one pinning site to the next. (**c**) Profile measured along the solid line in Fig. 5a. (**d**) Profiles measured along the purple and gray lines marked in Fig. 5b. Based on these images, non-conventional hysteresis loops of (**e**) homogeneous and (**f**) bamboo-like nanowires were depicted corresponding to the dashed lines in (**a**,**b**).

Figure 5a,b show the evolution of the magnetic contrast under *in-situ* applied magnetic fields for NWs with homogeneous and modulated diameter, respectively. For high magnetic fields, the homogeneous NW is saturated and thus the MFM signal exhibits the so called dipolar contrast (see the profile shown in Fig. 5a measured along the solid purple line). As the field is swept, the bright/dark contrast at the ends of the NW decreases gradually due to the reduction of the magnetostatic interaction between the tip and the sample. Such behavior is in agreement with the development of vortices at the ends of the nanowires -as predicted for NWs with diameters beyond a few tens of nanometers^29^. It is concluded from the simulations (SI. 4) that a vortex nucleates at one of the ends, right after the fields acquires a value below its saturation field; this vortex nucleates and grows in thickness, expanding towards the center. In the straight NWs, magnetization at the surface switches first followed by the core. This explanation is in good agreement with the experimental data shown in Fig. 5. At a certain value of the field, the magnetic contrast at the nanowire end is suddenly reversed when the closure structure becomes energetically unstable; a domain wall is thus depinned and propagates along the NW length resulting in a single large Barkhausen jump. Therefore, we can distinguish two critical values:(i) the first one, marked with a dashed arrow in Fig. 5, corresponds to the region where we are able to measure the enlargement of the vortex; (ii) the second one, marked with a bold arrow, where we see a single Barkhausen jump without intermediate configuration due to the quick propagation of the domain wall, 6 orders of magnitude beyond the scanning speed.

These images allow us to obtain non-conventional hysteresis loops of individual nanowires. In Fig. 5e the evolution of the contrast in one ends of the NW is depicted. MFM data in Fig. 5a,c suggest a bistable behavior where either positive or negative remanent configuration is possible. Notice that, in principle, DWs can nucleate simultaneously in both ends. However, the local geometry of each particular NW determines whether one or two DWs are involved in the magnetization reversal^30^. Slight differences in the critical fields between different nanowires are expected due to small morphological differences amongst them.

Unlike homogeneous nanowires, the measurements corresponding to bamboo NWs outstandingly show the intermediate pinning of the DW in specific diameter modulation sites near the ends of the NW (see the evolution of the dark contrast in the image corresponding to the branch from +45 to −45 mT in Fig. 5b). It should be noticed that the contrast at the enlarged diameter regions remains even at the highest applied field (see Fig. 5b). At a critical field, a DW depins and propagates along the NW. It is important to emphasize that at the diameter modulations the MFM contrast is reversed as the DW passes through, in good agreement with the micromagnetic simulations presented above. In the following image -corresponding to the branch from −45 to +45 mT-, we observe even more clearly that the DW stops in two identified modulated sites giving rise to small jumps in the hysteresis loop until finally reaching the far end of the NW. Since the driven parameter, the magnetic field, is sweeping continuously, after the first depinning, the applied magnetic field is much higher that the critical field i.e. the domain wall speed increases^31^,^32^. Thus, it is demonstrated that in these bamboo-like NWs the pinning at intermediate sites is a metastable configuration.

Small variations in the DW propagation and asymmetries are measured in successive field cycling for the same nanowire which evidences how critical the data acquisition speed might be in these measurements. We must keep in mind that the scanning speed of the MFM is around 100 microns per second, much smaller than the propagation velocity.

The normalized hysteresis loops of the modulated NW -shown in Fig. 5f- were obtained from the MFM image in Fig. 5b (measuring along the dashed lines).

However bamboo-like NWs present several intermediate configurations and therefore, the raw data from one of the nanowire ends is not representative of the whole NW. To obtain a conventional hysteresis loop a reconstruction procedure is needed. By using these results, we have calculated an average hysteresis loop for the modulated NW that allows us to give an average coercive value of 25 mT^33^. See Supplementary information III- for more details.

### Domain Wall pinning

Coming back to the data shown in Fig. 3, in some cases, strong black-white contrast is found, not only at the ends, but also in the middle part of the nanowire. Such features observed in remanence could be ascribed to the existence of domain walls pinned at those positions.

**Figure 6:**
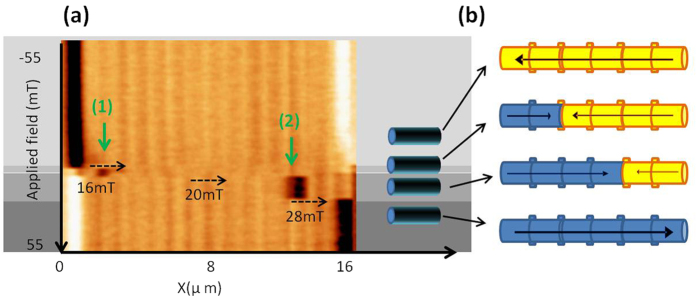
(**a**) Non standard MFM image of a NW where a DW is pinned. The DW “jumps” from one modulation to another under the externally applied magnetic field. The sketches in (**b**) illustrate the four different configuration observed in the MFM measurement.

Several measurements were performed on nanowires presenting this strong contrast at certain position along the length. Although it was found out that in some cases, the strong contrast is originated by an intense stray field (Supplementary Information IV), in other cases (see Fig. 6), it was proven not only that a DW is pinned, but we even succeeded to move the position of the domain wall from one pinning site to another.

Figure 6 shows the non-standard MFM image measured in one of those NWs. The magnetic field was swept between +/−70mT although here only the region from −55 to 55 mT is shown. In the image shown in Fig. 6, we observed the pinning of domain walls in different regions of the NW and at different magnetic fields. A segment on the left side begin their magnetization reversal at 16 mT, moreover, at 20 mT the domain wall jumps to the right and finally, the reversal of the whole nanowire does not occur until 28 mT are applied. Notice that, as expected, these NWs with pinned domains, present slightly magnetically harder behavior than the single domain bamboo-like NWs.

Consequently, making use of this advanced technique we can track the movement of the wall along the NW, and quantify the necessary field to move the DW and the exact position at which it will be pinned. This is a necessary feature for spintronic devices, which makes these bamboo-like nanowires promising candidates to be used in applications.

## Conclusions

Two different kinds of ferromagnetic nanowires -grown by electrodeposition in alumina templates- were studied: homogeneous in diameter and bamboo-like NWs. In the remanent state, both kinds of NWs present nearly single domain configuration, however, the magnetic configuration in the modulated NWs exhibits strong stray fields at the enlarged diameter positions.

Nevertheless, the two samples show different magnetization reversal processes. The MFM data reveals that while homogeneous NWs present an abrupt magnetization reversal through the depinning and propagation of a single DW, a metastable intermediate pinning has been measured in most of bamboo-like NWs. By using the non-standard variable field MFM technique we have been is able to image a local hysteresis loop and accurately determine the coercive fields of each individual NW that is takes a value of around 20–25 mT.

Finally, we have identified certain nanowires where it is possible to control the DW pinning by applying external magnetic fields. This process was imaged and quantified by Magnetic Force Microscopy.

## Methods

### Sample preparation

The bamboo-like nanowires were produced using the self-assembled pores of alumina templates obtained by pulsed hard anodization in oxalic aqueous solution (0.3M) containing 5 vol.% ethanol at a constant temperature of 0 °C^34^,^35^. In a first step a constant voltage of 80 V was applied for 900s to produce a protective aluminum oxide layer at the surface of the disc, which avoids breaking or burning effects during subsequent hard-pulse anodization^36^,^37^. Afterwards, the voltage is slowly increased (0.08 V/s) to 140 V and kept constant for 600 s, which ensures the parallel alignment of the nanochannels. The modulated nanopores were produced by periodically applying pulses of 140 V and 80 V for 30 and 10 s, respectively. The pulses were repeated 30 times to guarantee a total length of the modulated pores of few tens of microns. The wires were grown into the alumina pores by electrodeposition from a sulfate-based electrolyte^38^. The resulting periodically modulated pores are formed by 800 nm long segments, 150 nm in diameter, separated by much shorter segments, few tens of nanometer long, 170 nm in diameter, forming a bamboo-like structure as observed in Fig. 1. The center-to-center inter-pore distance is kept constant at 320 nm. After dissolving the alumina, the NWs are kept in ethanol and a drop of the solution is placed onto a silicon substrate.

### Magnetic Force Microscopy measurements

A scanning force microscope from Nanotec Electronica has been used to perform all the measurements, together with Nanosensors PPP-MFMR microchips. Amplitude modulation method was performed and the phase-locked loop (PLL) was enabled to track the resonance frequency of the oscillating cantilever. For this reason, the magnetic signal output is given in Hz.

### Micromagnetic Simulations

A 780 nm long cylinder has been simulated, with a diameter of 150 nm. To study the effect of the modulation, a broadening in the diameter has been included in the middle of the nanowire, reaching a maximum diameter of 170 nm. The modulation is a diameter enlargement of triangular shape with a base of 20 nm. Regarding the characteristics of the material, it was assumed that FeCoCu lacks of crystalline anisotropy (K_1_ = K_2_ = 0) and magnetization and exchange coupling constant were assumed to be M_s_ = 14 × 10^5^ A/m and A = 10.7 × 10^−12^ J/m respectively. The cubic cell size was chosen 2.5 nm, to be below the exchange length, which is approximately 3 nm for this material.

## Additional Information

**How to cite this article**: Berganza, E. *et al*. Domain wall pinning in FeCoCu bamboo-like nanowires. *Sci. Rep.* **6**, 29702; doi: 10.1038/srep29702 (2016).

## Supplementary Material

Supplementary Information
