# Supplementary material for: Domain wall pinning in FeCoCu bamboo-like nanowires
Source: Sci Rep. 2016 Jul 11;6:29702. doi: 10.1038/srep29702 (PMC4942567; doi:10.1038/srep29702)
Supplement: Supplementary Information [file srep29702-s1.pdf]

Domain wall pinning in FeCoCu bamboo-like nanowires

Eider Berganza, Cristina Bran*, Miriam Jaafar, Manuel Vázquez and Agustina Asenjo.

Instituto de Ciencia de Materiales de Madrid, CSIC, Madrid, 28049, Spain

Supplementary information

I. Non-standard Variable Field MFM mode

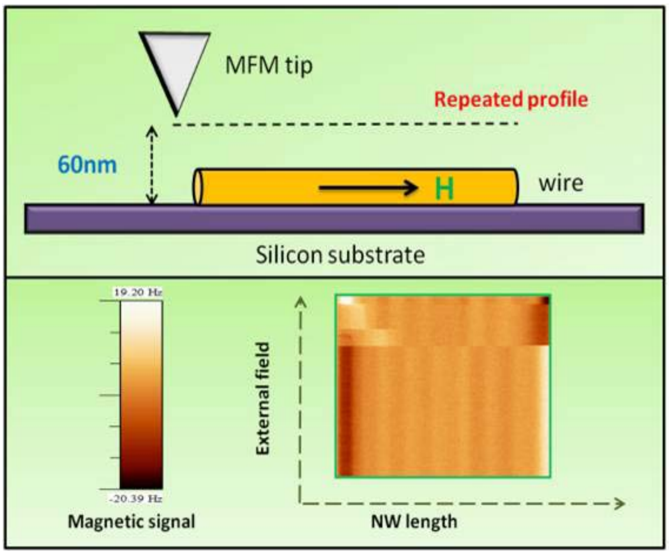

Fig. S1. (a) The followed procedure to obtain data with the non-standard MFM technique is illustrated. (b) Typical image obtained by using this mode.

Non-conventional MFM images (Figure 5a and Figure 5b) are obtained as follows: the tip is placed onto the desired nanowire and the scans are performed along the NW length at a typical distance of 60nm, as shown in Figure S1a. This scan line is repeated whereas the external applied magnetic field is swept between the maximum and the minimum of the magnetic field applied parallel to the nanowire axis.

As a result, in the MFM image in Figure S1b, X axis represents the length of the scanned line, Y the external applied magnetic field and Z the MFM signal for every position on the nanowire and every magnetic field value. The two images presented for the two samples (Figure 5a and Figure 5b), complete the full minor hysteresis loop, the first image corresponds to a field sweep from +35 to -35mT (+45 to -45mT in the case of Figure 5b), while the second corresponds to a field sweep beginning from -35mT to +35mT (-45mT to +45mT in the case of Figure 5b).

II. Simulated magnetization reversal of the straight nanowires.

In order to understand the decrease of contrast intensity present in figure 5a, at the ends of the nanowire as the field changes, simulations were carefully analyzed. A hysteresis loop was simulated and the spin configuration was recorded for different stages. The divergence of the magnetization was calculated with OOMMF and imaged in a gray scale. In the figure below, a transversal slice throw the middle of the cylinder is shown. As it can be seen, by decreasing the field from saturation, a vortex nucleates and expands towards the center of the NW. To illustrate this idea, the spin configuration is shown in the lateral slice for the marked positions.

This effect can be detected by advanced MFM modes as a decrease in the intensity, since MFM is a surface technique with high magnetic sensitivity. Nonetheless, the growth of the vortex is not detected in terms of lateral growth until it becomes very notable, due to the limitations in spatial resolution.

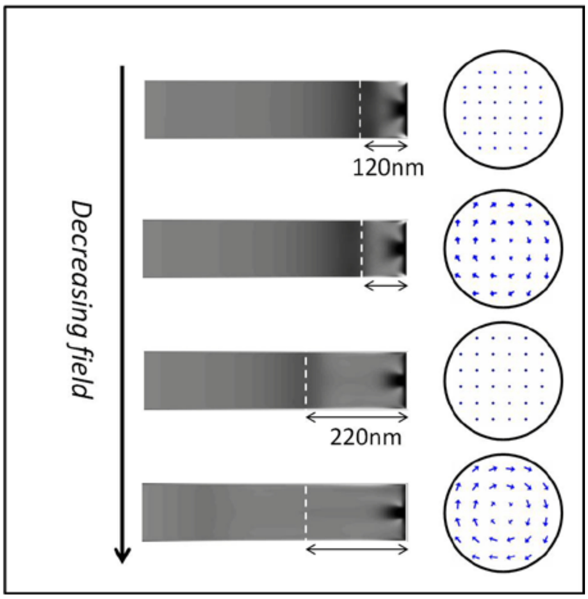

Fig. S2. Sequence showing the dynamical evolution of the vortex nucleated at one end of the NW. A segment of 500 nm has been represented.

III. Reconstruction of the hysteresis loops

We have evaluated the changes of the magnetic moment along the axis associated to the jumps as shown in the Fig. S3. In this simple model the initial and final magnetization values are considered -1 and 1 respectively, and the values from the configurations in between are calculated from purely geometrical considerations.

The hysteresis loops obtained when we plot the profiles obtained from the MFM data are similar to the conventional hysteresis loops which quantify the magnetization in the NW axis as a function of the applied field. Nevertheless, we have to keep in mind that MFM is sensitive to the stray field of the sample, in the out of plane direction. This means that, especially in the case of the diameter modulated NWs, although the

hysteresis curves might seem similar to the conventional, the size of the Barkhausen jumps is not real. Further calculus need to be performed, since a single profile at the edge gives only information of the local changes in the magnetization.

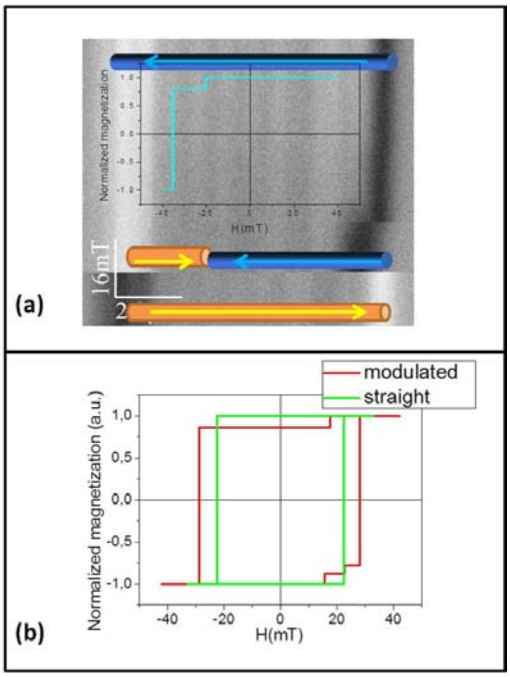

Fig. S3. (a) The normalized magnetization is calculated for each configuration using geometrical considerations. (b) Reconstructed hysteresis loops for both kinds of nanowires.

IV. REGIONS OF HIGH STRAY FIELD

In Fig. S4, we can find an example where a strong black-white contrast is found, not only at the edges, but in the middle of the nanowire. This intermediate contrast could lead us to the wrong conclusion that a DW has been pinned.

However, a deeper analysis of images obtained by using the mentioned non-standard variable field MFM technique reveals that in this case, the contrast does not move towards the end of the NW as expected. Instead, when the magnetization of the whole wire changes its direction, the area in the yellow circle reverses its contrast, as displayed in Fig. S4. This behavior cannot be explained with the existence of a pinned domain wall but rather with the existence of high stray field regions induced by defects.

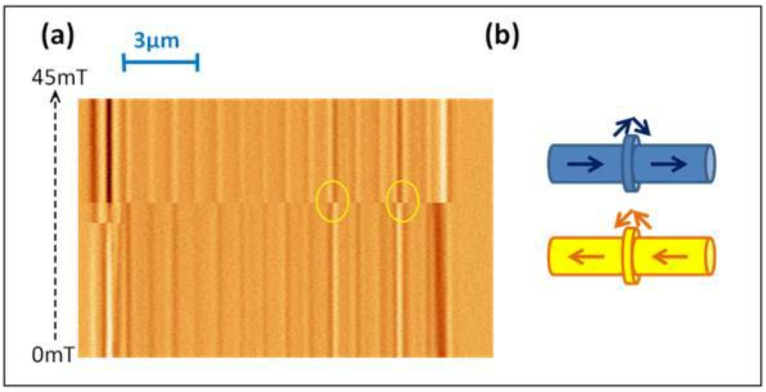

Fig. S4. (a) Non standard MFM image of a NW which seems to have a pinned DW in its remanent state. It is demonstrated that the strong contrast spots, inside the yellow circle, cannot be ascribed to a DW, as it is illustrated in (b).
